# Antigen of 49.6-kDa subunitpili protein of *Helicobacter pylori* as a potential biomarker for early and rapid detection of the infection

**DOI:** 10.14202/vetworld.2019.769-773

**Published:** 2019-06-10

**Authors:** Hamong Suharsono, Zainul Muttaqin, I Wayan Masa Tenaya, Kadek Karang Agustina, Sumarno Retro Prawiro

**Affiliations:** 1 Laboratory of Biochemistry, Faculty of Veterinary Medicine, Udayana University, Denpasar, Indonesia; 2 Biomedical Research Unit, West Nusa Tenggara General Hospital, Lombok, Indonesia; 3 Animal Diseases Investigation Center, Denpasar, Indonesia; 4 Department of Veterinary Public Health, Faculty of Veterinary Medicine, Udayana University, Denpasar, Indonesia; 5 Laboratory of Microbiology, Medical Faculty of Brawijaya University, Malang, Indonesia

**Keywords:** 49.6-kDa pili protein, *Helicobacter pylori*, immunochromatographic test

## Abstract

**Background and Aim:**

*Helicobacter pylori* infection has been identified as a major cause of peptic ulcer diseases, including gastric and duodenal ulcers, gastritis, chronic and gastric carcinoma, and even gastric lymphoma. *In vitro* studies using Western blotting analysis, hemagglutination test, adherence inhibition assays, and immunocytochemical staining revealed that the 49.6-kDa subunit pili protein of *H. pylori* was considered an immunogenic protein. This study aimed to develop a serological diagnostic test using 49.6 kDa for detecting antibodies against *H. pylori* proteins in an early phase of the infection.

**Materials and Methods:**

An in-house immunochromatographic test (ICT) kit was developed and used to test a panel of sera sample obtained from a randomly selected symptomatic patient, in which 40 sera were *H. pylori* positive and 40 sera were *H. pylori* negative.

**Results:**

The results showed that ICT with 49.6 kDa as an antigen was highly sensitive and specific for detecting anti-*H. pylori* immunoglobulin G antibodies in human serum, with a high negative predictive value.

**Conclusion:**

The developed test could be used to exclude *H. pylori* infection in symptomatic patients.

## Introduction

Infection by *Helicobacter pylori* has been identified as a major cause of peptic ulcer diseases (gastric and duodenal ulcers), gastritis, chronic and gastric carcinoma, and even gastric lymphoma [1,2]. *H. pylori*, also known as a zoonotic agent, is isolated from cow, sheep, and goat [3], and its antigens were detected in the milk and feces of cows [4]. This is unique because *H. pylori* is the only bacterium known to cause gastric carcinoma [5-7].

Infectious *H. pylori* was classified as carcinogen group I for gastrointestinal cancer because it has various virulent factors, such as CagA, VacA, Urease, and ammonia, that are capable of triggering carcinogenesis [5,8-10]. The pathogenic properties of these bacteria were associated with its fimbrial adhesion (pili) [11], a protein found on the bacterial cell surface that plays a role as a bacterial virulence factor [12]. *In vitro* studies using Western blotting analysis, hemagglutination test, adherence inhibition assays, and immunocytochemical staining revealed that the 49.6-kDa subunit pili protein of *H. pylori* was immunogenic [13]. The ability of this protein in agglutinating mouse erythrocytes indicates that it was hemagglutinin in nature [13]. It was also confirmed that the protein possessed adhesion molecules that play a crucial role in the early phase of the pathogenesis when *H. pylor*i infiltrate into the epithelial cells of gastric [14-16].

Further study is required to investigate the diagnostic value of this protein in the early detection of infection by this microorganism. Most diseases present high cure rates only when detected early [17]. Therefore, early diagnosis is essential to reduce morbidity and mortality. In addition, early diagnosis of infectious diseases can prevent their development into epidemics. There are several methods to detect *H. pylori* infection [18,19]. Detection of *H. pylori* infection by using serological methods is considered to be the easiest, non-invasive approach, which does not require endoscopy to diagnose the infection [20,21]. This method requires only a few drops of blood, producing results in <5 min [22]. There are numerous methods available for the detection of anti-*H. pylori* immunoglobulin G (IgG), immunoglobulin A, and immunoglobulin M antibodies [23], which are present in the whole blood, serum, saliva, stool, and urine. The accuracy of the diagnostic markers varies from test to test and among sample types [24,25]. In the present study, we evaluated the performance of a new immunochromatographic test (ICT) kit using 49.6-kDa pili protein.

## Materials and Methods

### Ethical approval

This research was approved by the Ethical Commission for the Use of Animals in Research and Education of the Faculty of Veterinary Medicine, Udayana University, Indonesia with Ref. No. 284a/KE-PH/VII/2017.

### *H. pylori* strains

Three *H. pylori* Lombok isolates were provided by the Microbiology Laboratory, Biomedical Research Unit, West Nusatenggara General Hospital, which were isolated from the gastric antral biopsies of Sasak Lomboknese patients. The bacterium was first cultured using media Trypticase Soy Agar and Trypticase Soy Broth supplemented with 10% sheep blood, completed with supplement and IsoVitaleX™, and incubated at 37°C on the microaerophilic atmosphere [13].

### Isolation of 49.6-kDa subunit pili protein of *H. pylori*

Isolation of *H. pylori* pili was performed by the method of Sumarno *et al*. [26] with a slight modification. Bacteria pili were cut by using a pili bacterial cutter, which was carried out for 30 s at the speed of 5000 rpm and repeated for five times. Subsequently, the isolation of pili fraction by centrifugation of cutting result was done at 12,000 rpm at 4°C. The supernatant containing the bacterial pili was analyzed in sodium dodecyl sulfate-polyacrylamide gel electrophoresis. Protein of 49.6-kDa subunit pili was isolated from the gel by cutting alongside close to the protein positions, around 49.6 kDa. The isolated gel was sliced and inserted into the dialysis membrane soaked with phosphate-buffered saline (PBS). Subsequently, the protein of interest was electroeluted by placing the membrane in the negative electrode with current of 20 mA for 15 min. Total protein was measured using a method ofdetergent-compatible protein assay (Bio-Rad Laboratories Inc, USA), suspended to a concentration of about 10 ng/ml, and kept at −20°C until use [13].

### Sera panel from dyspepsia patients and vaccinated mice

#### Human sera

Eighty sera from *H. pylori*-positive and *H. pylori*-negative patients based on the culture and direct microscopic examination of mucosal gastric biopsy were used as panel sera. All sera of dyspepsia patients were provided by the Biomedical Research Unit, West Nusa Tenggara General Hospital, and stored at −20°C.

#### Mouse sera

One hundred healthy male balb/c mice (weight 18–22 g) were used in this study for producing animal sera. The experimental mice were grouped into 10 groups and fed with a portion of commercial food and water *ad libitum*. All mice were orally given culture of live *H. pylori*, they had been fasted one night. The *H. pylori* obtained from a patient with typical gastric ulcer was first cultured with Brain Heart Infusion media supplemented with 5% sheep blood, incubated at microaerophilic condition (10% CO_2_, 85% N_2_, and 5% O_2_) at 37°C for 48 h. The cells were then washed and suspended with sterile PBS at a concentration of 109 cells/ml. The 100 balb/c mice were divided into two groups of A and B, with 50 balb/c mice each. Each animal in Group A was orally infected with 109 cells/ml 3 times every 2 days, based on the method of Marchetti *et al*. [27] In contrast, all animals in Group B were used as control animals that were orally given sterile PBS containing no *H. pylori* culture. Blood samples were collected from both Groups A and B before they were orally infected with the bacterial culture, and 1, 2, and 3 weeks after the infection. The blood samples were collected from the tail and kept at room temperature (RT) for 2–5 h before the sera were collected and stored at −20°C until tested.

### Development of IgG *H. pylori* ICT

A standard ICT strip typically consists of five parts: Sample (polyester) pad, conjugation (polyester) pad, nitrocellulose (NC) membrane, absorption pad, andpolyvinyl chloride plastic backed card. All the pretreated parts were assembled sequentially on a plastic backing card with 2-mm overlap of each component. Antigens (49.6 kDa-sub-unit protein pili or secretory antigen [Ag]) were coated separately to serve as test line and goat anti-mouse IgG as control line [28]. Both antigens and antibodies were dispensed by XYZ dispensing system (BioDot Inc, USA) to NC membrane. The Protein A-colloidal gold (CG) conjugate was dispensed onto the polyester pad. After being dried at 37°C, the assembly was cut into a 5-mm-wide individual strip and then stored at the RT inside a sealed plastic bucket with a desiccant until used. When a serum sample is added onto the sample pad, the sample flowed from this component to the absorption pad on the membrane surface by capillary action. Once the sample reached specific positions, the antigen in the sample reacted with the conjugates (labeled with CG). The residual sample continues to move forward and be absorbed in the absorption pad. After the reaction was completed, two lines appeared on the strip: The C-line (control line to confirm whether the strip is valid) and the T-line (test line used to judge the detection results) [29].

### Detection of antibodies in patient serum

The ICT cards were removed from the foil pouch and placed on a flat, dry surface. A drop (20–30 μl) of serum was applied on to the sample well. Then, two drops of the buffer were added on to the sample well. As the test began to work, purple color was seen moving across the result window at the center of the test disk. Test results were interpreted within 20 min. The presence of two-color bands, “T” and “C,” meant that the test was positive, whereas the presence of only one band (only on “C”) was interpreted as negative. If no band or a single band only on “T” was formed after 20 min, the result was considered invalid, and the experiment was repeated [30].

## Results

### Demographic and geographic characteristics

We used 80 sera from dyspepsia patients undergoing an endoscopic examination at the West Nusatenggara Province General Hospital. Demographic characteristics of gastric disease patients are shown in Table-1.

**Table-1:** Demographic characteristics.

Characteristics	n (%)
Sex	
Male	52 (65.0)
Female	28 (35.0)
Age (years)	
<30	4 (5.0)
30–50	60 (75.0)
>50	16 (20.0)
Ethnicity	
Sasak (Lomboknese)	66 (82.5)
Balinese	12 (15.0)
Other	2 (2.5)
Endoscopy diagnosis	
Chronic gastritis	58 (72.5)
Gastric or DU	14 (17.5)
GC	8 (10.0)

GC=Gastric cancer, DU=Duodenal ulcer

The average age of the patients was 48.22 years. The representation per age group was 4 (5.0%) patients under 30 years; 60 patients (75.0%) between 30 and 50 years; and 16 patients (20.0%) >50 years. Considering the age, most of the patients were adult; hence, the diagnosis resulted in chronic *H. pylori* infection [31].

### Antigen 49.6-kDa subunit pili protein of *H. pylori*

**Figure-1:**
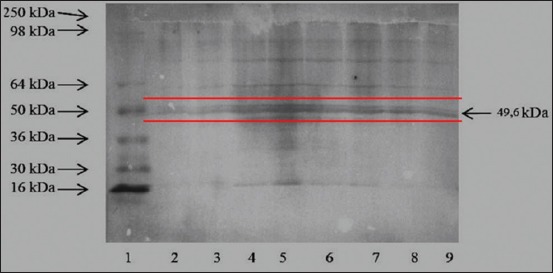
Western blot analysis of protein subunit pili protein from sequential cutting *H. pylori* against rabbit polyclonal anti-*H. pylori* shows that the polyclonal antibodies react strongly with subunit pili protein with a molecular weight of about 49.6 kDa, compared to other protein components. Lanes: 1. Molecular weight marker, 2–9: Protein fractions.

The result of Western blotting analysis for detecting the 49.6-kDa subunit pili protein of *H. pylori* is shown in Figure-1.

### Comparison of IgG *H. pylori* detection results

The presence of specific antibodies in mice which had been infected orally using the culture of live *H. pylori* was demonstrated using a standard ICT. Strong and specific reaction was observed when the subunit protein pili Ag reacted with the mouse sera 3 weeks after the infection. However, a negative reaction was observed when the sera were reacted with the secretory Ag (Figure-1). The prevalence of the serological reaction depended on the time after the infection; the highest prevalence of 96% was observed 3 weeks after the initial infection (Table-2).

**Table-2:** Comparison of ICT result between subunit pili Ag and secretory Ag to detect the presence of IgG anti-*Helicobacter pylori* prepared from vaccinated mouse sera.

Testing result	Post-infected mouse sera (n=50)		

	1 week	2 weeks	3 weeks
Secretory Ag ICT			
Positive	0 (0)	2 (4)	39 (78)
Negative	50 (100)	48 (96)	11 (22)
Subunit pili Ag ICT			
Positive	0 (0)	17 (34)	48 (96)
Negative	50 (100)	33 (66)	2 (4)

IgG=Immunoglobulin G

Strong positive reaction in B (subunit protein pili Ag) was observed in sera of mice 3 weeks after the infection. No reaction was observed with the same sera in B (secretory Ag) [32].

The presence of specific antibodies against secretory Ag and subunit pili Ag in dyspepsia patients undergoing endoscopic was demonstrated using *in vitro* studies employing ICT. The subunit protein pili reacted 37 out of the 40 sera from an infected human with a specificity of 92.5% (true-positive detection) and detected only 1 out of the 40 sera (2.5%) (false-negative reaction) from the non-*H. pylori*-infected patients. Although the ICT secretory Ag reacted with sera from an infected human with a specificity of 95%, this protein also reacted with 5 out of the 40 sera (12.5%) (false negative) from the non-*H. pylori*-infected patients, suggesting that this protein was less specific than the 49.6 kDa-sub-unit pili protein of *H. pylori* (Table-3).

**Table-3:** ICT result of secretory Ag and subunit pili Ag to detect the antibody of *H. pylori* in panel sera of *H. pylori.*

Testing result	*H. pylori* (+) (n=40)	*H. pylori* (−) (n=40)
ICT secretory Ag		
Positive	38	5
Negative	2	35
ICT subunit pili Ag		
Positive	37	1
Negative	3	39

*H. pylori=Helicobacter pylori*.

## Discussion

**Figure-2:**
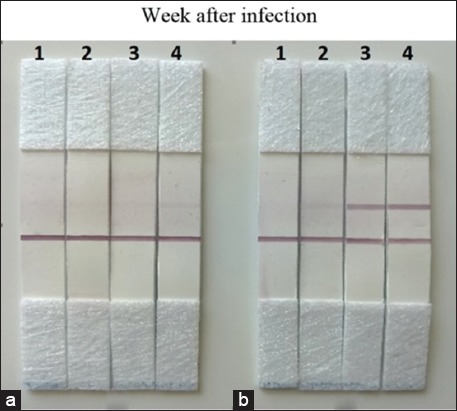
Test result of the different strip to detect antibody against *H. pylori* antigen in *H. pylori*-infected mouse sera: (a) Secretory Ag and (b) subunit protein pili Ag.

*H. pylori* infection can be diagnosed using either invasive or non-invasive approaches [33]. Among the non-invasive approaches, serological techniques are widely used because they are cost-effective, simple, and quick [22,34]. However, it is unreliable to differentiate between active and previous infections [18]. Due to this condition, it was considered essential to develop a serological test to evaluate the progression of infection, especially in conjunction with eradication therapy. Therefore, a new non-invasive diagnostic test developed based on the detection of *H. pylori* IgG in this study was found to be a reliable test for detecting antibodies against *H. pylori* proteins. Using ICT test, both the 49.6 kDa-sub-unit pili protein of *H. pylori* and ICT secretory Ag were found to be sensitive in detecting the presence of antibodies in vaccinated mice, from the 2^nd^ week after vaccination. However, the 49.6 kDa-sub-unit pili protein was more sensitive than ICT secretory Ag. The first antigen demonstrated 34% and 96% of positive reaction at the 2^nd^ and 3^rd^ weeks after the infection. Meanwhile, the second protein showed positive reaction of 4% and 78% at the 2^nd^ and 3^rd^ weeks, respectively (Figure-2). The use of the prepared antigen to test sera from dyspepsia patients undergoing endoscopy using the ICT test showed that it reacted 37 out of the 40 sera from an infected human with a specificity of 92.5% and detected 1 out of the 40 sera (2.5%) from the non-*H. pylori*-infected patients. Although the ICT secretory Ag reacted with sera from an infected human with a specificity of 95%, this protein also reacted with 5 out of the 40 sera (12.5%) from the non-*H. pylori*-infected patients, suggesting that this protein was less specific than the 49.6-kDa sub-unit pili protein of *H. pylori*.

## Conclusion

In this study, a specific 49.6-kDa sub-unit pili protein was found to be a potential biomarker for the early and specific detection of *H. pylori* infection. From two antigens compared in this study, both secretory Ag ICT and sub-unit pili Ag ICT recognized specific antibodies that were prepared from mice experimentally infected with live *H. pylori* and from dyspepsia patients undergoing endoscopy. However, the 49.6-kDa sub-unit pili protein of *H. pylori* was found to be more sensitive than the secretory Ag, which could detect the presence of targeted antibodies in the 2^nd^ week after vaccination in mice. Moreover, the 49.6-kDa sub-unit pili protein demonstrated true-positive detection of 92.5% and true-negative detection of 97.5% in human sera. In contrast, the secretory Ag ICT showed true-positive and true-negative detection of 95% and 87.5%, respectively. It was concluded that a feasible serological test developed in this study, using a specific 49.6-kDa sub-unit pili protein of *H. pylori*, was considered to be an important antigen used in serological tests for monitoring dyspepsia patients undergoing endoscopy; therefore, it was recommended to apply this protein in serological test for the detection of *H. pylori* infection in humans.

## Authors’ Contributions

HS: Designed and managed this research, did laboratory works, and wrote the manuscript; ZM: Collected samples and wrote the manuscript; IWMT: Did laboratory works, analyzed the data, and wrote the manuscript; KKA: Analyzed the data and wrote the manuscript; SRP: Designed the research, analyzed the data, and wrote the manuscript. All authors read and approved the final manuscript.
